# Identification and genetic analysis of cancer cells with PCR-activated cell sorting

**DOI:** 10.1093/nar/gku606

**Published:** 2014-07-16

**Authors:** Dennis J. Eastburn, Adam Sciambi, Adam R. Abate

**Affiliations:** Department of Bioengineering and Therapeutic Sciences, California Institute for Quantitative Biosciences, University of California, San Francisco, CA 94158, USA

## Abstract

Cell sorting is a central tool in life science research for analyzing cellular heterogeneity or enriching rare cells out of large populations. Although methods like FACS and FISH-FC can characterize and isolate cells from heterogeneous populations, they are limited by their reliance on antibodies, or the requirement to chemically fix cells. We introduce a new cell sorting technology that robustly sorts based on sequence-specific analysis of cellular nucleic acids. Our approach, PCR-activated cell sorting (PACS), uses TaqMan PCR to detect nucleic acids within single cells and trigger their sorting. With this method, we identified and sorted prostate cancer cells from a heterogeneous population by performing >132 000 simultaneous single-cell TaqMan RT-PCR reactions targeting vimentin mRNA. Following vimentin-positive droplet sorting and downstream analysis of recovered nucleic acids, we found that cancer-specific genomes and transcripts were significantly enriched. Additionally, we demonstrate that PACS can be used to sort and enrich cells via TaqMan PCR reactions targeting single-copy genomic DNA. PACS provides a general new technical capability that expands the application space of cell sorting by enabling sorting based on cellular information not amenable to existing approaches.

## INTRODUCTION

The analysis of individual cells from a heterogeneous population can reveal information relevant to human health and disease unobservable when studying the entire population in bulk (1–4). Examples of heterogeneous cell populations that have a profound impact on human health include circulating tumor cells in blood, primary tumors, virally infected cell populations, niche residing stem cells and the immune system. Due to the potential for rare but biologically important cell types in these examples, obtaining meaningful information on these populations necessitates tools capable of single-cell analysis with high-throughput.

Perhaps the most effective tool for analyzing large numbers of single cells is fluorescence-activated cell sorting (FACS). Its ability to combine extremely high-throughput processing with single cell analysis is unparalleled in biological research and has made it an indispensable tool in the life science lab. Nevertheless, FACS suffers from several limitations that impede its use in many circumstances. It requires antibodies that bind specifically to the target cell; often, antibodies are not immediately available and generating new ones is laborious, expensive and sometimes ineffective. The protein of interest must also be localized on the cell surface where it is accessible to the antibody; if not, cells must be fixed and permeabilized, a process that can damage nucleic acids and prohibit additional analysis (5). The sensitivity of antibody labeling is also limited, making it difficult to detect proteins expressed at low levels. Most importantly, antibodies are unable to differentiate between cells based on their nucleic acids, including genomic mutations, non-coding RNAs and unique mRNA splice variants, precluding FACS sorting based on these important biomarkers. Fluorescence *in situ* hybridization-flow cytometry (FISH-FC) combines the throughput of FACS with the ability to label, and thereby detect, nucleic acids within single cells; however, it also requires chemical fixation, often yields low signals that are difficult to detect with FACS, and is unreliable for detecting many important cellular nucleic acids, including single nucleotide polymorphisms (SNPs) and microRNAs (6).

Polymerase chain reaction (PCR) is an extremely sensitive and accurate method for characterizing the nucleic acids of cells. PCR assays can be rapidly targeted to detect nearly any nucleic acid biomarker within a cell, and the process does not destroy nucleic acids, permitting additional analysis with qRT-PCR, microarrays or next-generation sequencing. However, applying PCR to the analysis of large populations of single cells, despite its clear potential, is challenging, because existing methods are laborious, consume extensive reagent and also lack the throughput necessary to analyze populations of biologically-relevant size, or in which the target cell is rare (2,3,7). To enable robust sorting of single cells based on nucleic acids, new methods are needed that combine the throughput and sorting of FACS with the sensitivity and generality of PCR.

In this report, we present a new cell sorting technology that can robustly detect nucleic acids within single cells using PCR and sorts based on this information. In our method, which we dub PCR-activated cell sorting (PACS), individual cells are encapsulated in microfluidic droplets and subjected to TaqMan PCR (8,9). Fluorescent TaqMan probes specific to the biomarkers of interest produce a detectable signal in the droplet when the target is present, allowing us to recover positive cell lysates by sorting the encapsulating droplets. Compared to FISH-FC, PACS can identify all nucleic acids in a cell detectable with TaqMan PCR, requires minimal assay optimization and, as we show, minimally perturbs RNA and DNA, allowing downstream sequencing of sorted populations. In addition, it is ultrahigh-throughput, allowing analysis and sorting of hundreds of thousands of single cells. These features make PACS complementary to FACS, enabling the analysis of biomarkers undetectable with antibodies and well suited for the study of rare or unique cell populations intractable with current methods.

## MATERIALS AND METHODS

### Cell culture and staining

Human DU145 prostate cancer and Raji B-lymphocyte cell lines were cultured in RPMI 1640 supplemented with 10% FBS, penicillin and streptomycin at 37°C with 5% CO_2_. Prior to cell staining, Raji cells were pelleted and washed once in phosphate buffered saline (PBS). Adherent DU145 cells were trypsinized prior to pelleting and washing. Cells were stained in 1 ml Hank's balanced salt solution (HBSS) with 2 μM Calcein Violet AM or Calcein Green AM for 30 min at room temperature. Following staining, cells were washed with PBS and then resuspended in PBS that was density matched with OptiPrep solution prior to encapsulation in microfluidic droplets.

To generate cell suspensions with known ratios of cell types, we performed cell counting and viability analysis on individual cell lines. This was done by combining a 10 μl aliquot of each cell type with an equal volume of trypan blue and placing the mixture into a chamber slide. Live cell numbers were determined by reading the chamber slides with the Countess Automated Cell Counter (Invitrogen).

### Fabrication and operation of microfluidic devices

The poly(dimethylsiloxane) (PDMS) devices were fabricated using standard soft lithographic techniques and operated as previously reported (8,10). Fluid flow was regulated via computer-controlled syringe pumps (NewEra) connected to the PDMS devices with polyethylene tubing. Fluorinated oil (FC40) with 5% PEG-PFPE amphiphilic block copolymer was used to generate the initial microdroplet emulsion (11). Lysis buffer (100 mM Tris pH 8.0, 2% Tween-20, proteinase K 1.5 mg/ml) was introduced at the time of cell encapsulation using a co-flow drop maker to prevent premature rupture of cells (8).

### Microdroplet TaqMan RT-PCR

Amplification primers for the vimentin RT-PCR reactions were as follows: Primer1 5′-GTGAATCCAGATTAGTTTCCCTCA-3′, Primer2 5′-CAAGACCTGCTCAATGTTAAGATG-3′; The sequence of the vimentin TaqMan probe was: 5′-HEX/CGCCTTCCA/ZEN/GCAGCTTCCTGTA/IABkFQ-3′. TaqMan reaction primers and probes were purchased as a pre-mixed assay from Integrated DNA Technologies (IDT). Superscript III reverse transcriptase and Platinum Taq DNA polymerase (Invitrogen) were used for the microdroplet single-cell TaqMan reactions. Thermocycling conditions were 50°C for 15 min followed by 93°C for 2 min and 45 cycles of: 92°C, 15 s and 60°C, 1 min. Thermocycled droplets were either imaged on a fluorescent microscope to confirm specificity of TaqMan reactions or transferred to a 1 ml syringe and reinjected into a microfluidic droplet sorter.

### Ultrahigh-throughput detection and sorting of droplets

Droplets were sorted dielectrophoretically using custom LabVIEW code controlling an FPGA card (National Instruments) (12,13). Two lasers (405 and 532 nm) were focused onto the channel upstream of the sorting junction, allowing the droplets to be scanned for fluorescence. The FPGA card analyzed the emitted fluorescence measured with spectrally-filtered PMTs (Hamamatsu Photonics) and outputted a train of 1 kV, 30 kHz pulses to the microfluidic electrode via a high voltage amplifier (Trek) to direct appropriate droplets into a collection channel. Droplets that had merged prior to sorting were measurably large and automatically discarded. During detection, the average HEX and calcein violet fluorescence of each droplet were recorded and plotted with MATLAB code. To quantify sorting efficiency, sorted droplets were analyzed with MATLAB code which identified droplets based on their circular boundary in brightfield images and then measured their fluorescence in the associated epifluorescence images.

### SRY TaqMan PCR assay

The Y chromosome detection assay was designed based on the sequences from a previously published *SRY* gene TaqMan probe and primer set (14). Human male and female mixed cell suspensions were generated using DU145 prostate cancer cells and female HEK293 cells. Cell lines were individually stained with either calcein green or calcein violet prior to mixing roughly equivalent ratios of each cell type. The single-cell PCR reactions were performed similarly to the vimentin RT-PCR TaqMan assay; however, Superscript III reverse transcriptase was omitted from the reaction. As an additional measure to ensure assay specificity for genomic DNA, RNase A was also added to the reaction.

### DNA sequencing of PACS-sorted genomic DNA

Following collection of sorted droplets, emulsions were broken using perfluoro-1-octanol and the aqueous fraction was diluted in 10 mM Tris pH 8.0. The aqueous layer containing the pooled cellular lysate was then purified using a DNeasy Blood and Tissue Kit (Qiagen). *CDKN2A* and *RB1* were PCR amplified from gDNA isolated from both presorted and vimentin sorted emulsions. Amplicons were analyzed on agarose gels and extracted with a Qiagen Gel Extraction kit. Fifty nanogram of gel extracted DNA was sent for Sanger sequencing and the data was analyzed on 4Peaks sequencing and chromatogram analysis software.

For next-generation sequencing, 1 ng of each amplicon was subsequently used for sequencing library preparation using the Nextera XT library kit (Illumina). Sequencing was done on a HiSeq2500 sequencer with 50 bp reads. Each library was indexed with a barcode and reads were automatically partitioned post sequencing. Next-generation sequence analysis was performed using the Galaxy web-based platform (15). The workflow consisted of quality checking sequence data with FASTQ Groomer, mapping the data to a reference sequence with Bowtie, converting the mapped data to a SAM file and then generating a pileup of the sequence data (16–18). Pileup data were analyzed for the presence of Raji or DU145-specific SNPs at the relevant positions. More than 15 000 base reads were analyzed for *RB1* and *CDKN2A* SNP positions from both the presorted and vimentin-positive PACS amplicon libraries.

### Quantitative RT-PCR analysis of PACS-sorted RNA

After breaking the emulsions with perfluoro-1-octanol, aqueous fractions from the droplets were collected and purified on an RNA binding column (Qiagen). Following elution, the extracted RNA was analyzed with TaqMan RT-PCR assays (Integrated DNA Technologies). Amplification reagents were from the SuperscriptIII One-Step RT-PCR System (Invitrogen). Three replicates were performed for each reaction and GAPDH was used to verify that equal amounts of RNA were used in each of the CD9 reactions. Quantitative reactions were carried out using an MX3005p Real-Time PCR System (Stratagene). Normalized fluorescence values from the instrument were plotted using Prism software. For control reactions, total RNA was first isolated from Raji and DU145 cell lines using an RNeasy purification kit (Qiagen). Eighty nanograms of total RNA was used as template for each qRT-PCR reaction. Differences in expression levels were calculated using normalized Ct values obtained from the amplification plots.

## RESULTS

### PACS workflow

**Figure 1.**
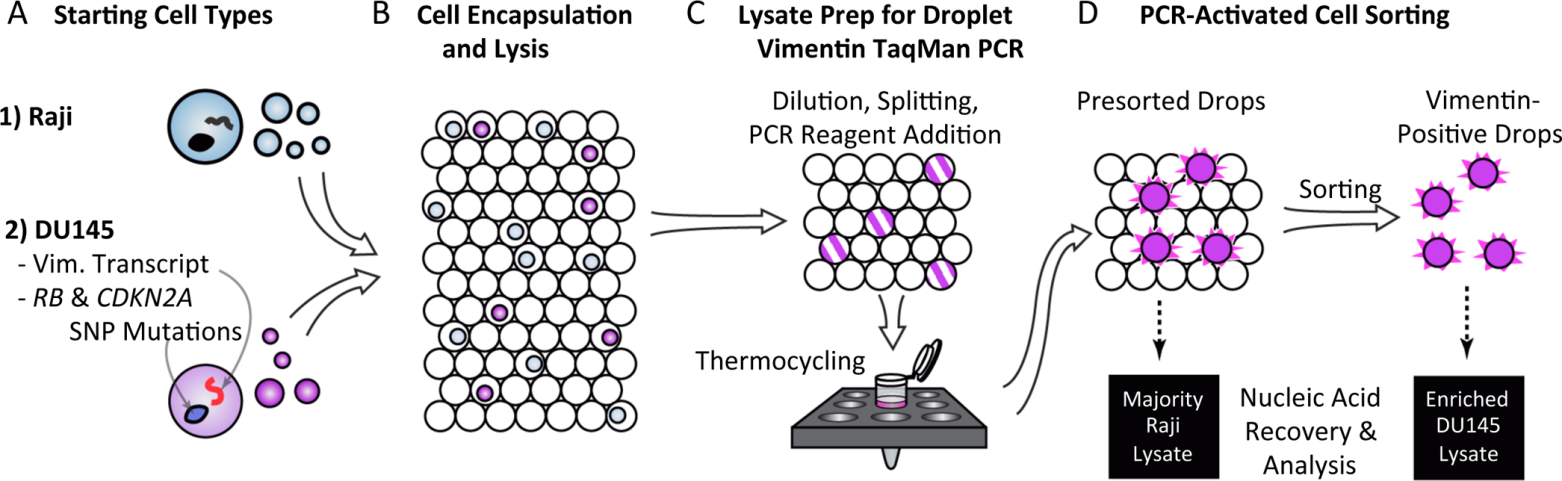
Workflow for PCR-activated cell sorting. (**A**,**B**) Raji and DU145 cells are isolated into aqueous microdroplets in an oil-based emulsion and lysed. Only DU145 cells express vimentin mRNA and have genetic mutations in *RB1* and *CDKN2A* genes. (**C**) A microfluidic chip then processes the cells, readying the lysate for PCR. Single-cell TaqMan PCR reactions targeting vimentin mRNA are thermocycled and droplets are sorted based on positive TaqMan probe fluorescence. (**D**) Following microfluidic droplet sorting, the cell lysate can be recovered for downstream nucleic acid analysis.

Sorting cells using TaqMan RT-PCR as an assay readout has never before been demonstrated, due both to the difficulty of preparing and handling stable, single-cell containing droplets and also lysate-mediated inhibition of the reaction in subnanoliter volumes (7,19,20). The technical advance key to enabling PACS has been our development of a robust microfluidic workflow that maintains compartmentalization of single-cell lysates at all times while also overcoming mammalian cell lysate-mediated inhibition of PCR (8). To perform PACS, cells are first encapsulated in aqueous droplets with Tween-20 and proteinase K lysis reagents (Figure 1A,B). The compartmentalized lysates of single cells are then diluted by droplet merger and TaqMan RT-PCR reagents added by droplet picoinjection (Figure 1C). The droplets, now prepared for efficient, uninhibited single-cell TaqMan RT-PCR, are collected and thermocycled for amplification to identify cells expressing target transcripts. We have previously reported using this technique to achieve a single-cell RT-PCR throughput of 47 000 mammalian cells (8). Additionally, we have shown that our approach is highly specific, enabling the unambiguous detection of Raji cells in a mixed suspension containing PC3 cancer cells. To enable lysate recovery of cells positive for the nucleic acid biomarker, we now also implement sorting of the microfluidic droplets based on the presence of the fluorescent signal produced from the TaqMan reaction (Figure 1D) (12,13). TaqMan positive droplets containing cell lysate of interest are collected and the nucleic acids extracted for downstream analysis.

### Vimentin-based prostate cancer cell detection

TaqMan RT-PCR assays offer single molecule sensitivity and can be precisely targeted to a wide variety of gene transcripts, making them ideal for distinguishing between and sorting cells (8,21). To demonstrate the utility of PACS-based TaqMan RT-PCR to identify specific cells in a heterogeneous population, we targeted expression of vimentin in DU145 prostate cancer cells spiked into Raji B-lymphocyte-derived cells. Vimentin is an intermediate filament protein known to participate in epithelial-to-mesenchymal transitions and can serve as a biomarker for some cancer cell types (22). It is expressed at an approximately 380-fold higher level in DU145 cells than in Raji cells based on bulk qRT-PCR analysis (Supplementary Figure S1) (23,24). The Raji cells thus serve as both an essential control for the specificity of the TaqMan reactions and as a more abundant ‘background’ cell type to assess the effectiveness of PACS enrichment of DU145 cells.

To measure the specificity and detection rate of PACS sorting based on vimentin expression, we labeled DU145 cells with calcein violet and Raji cells with calcein green viability stains. The vimentin TaqMan probe was labeled with HEX fluorescent dye having minimal spectral overlap with the calcein dyes. This three-color detection strategy enabled us to correlate vimentin mRNA detection with the presence of a specific cell type, and thereby measure the rate at which our droplet TaqMan RT-PCR was able to correctly distinguish between cells.

**Figure 2.**
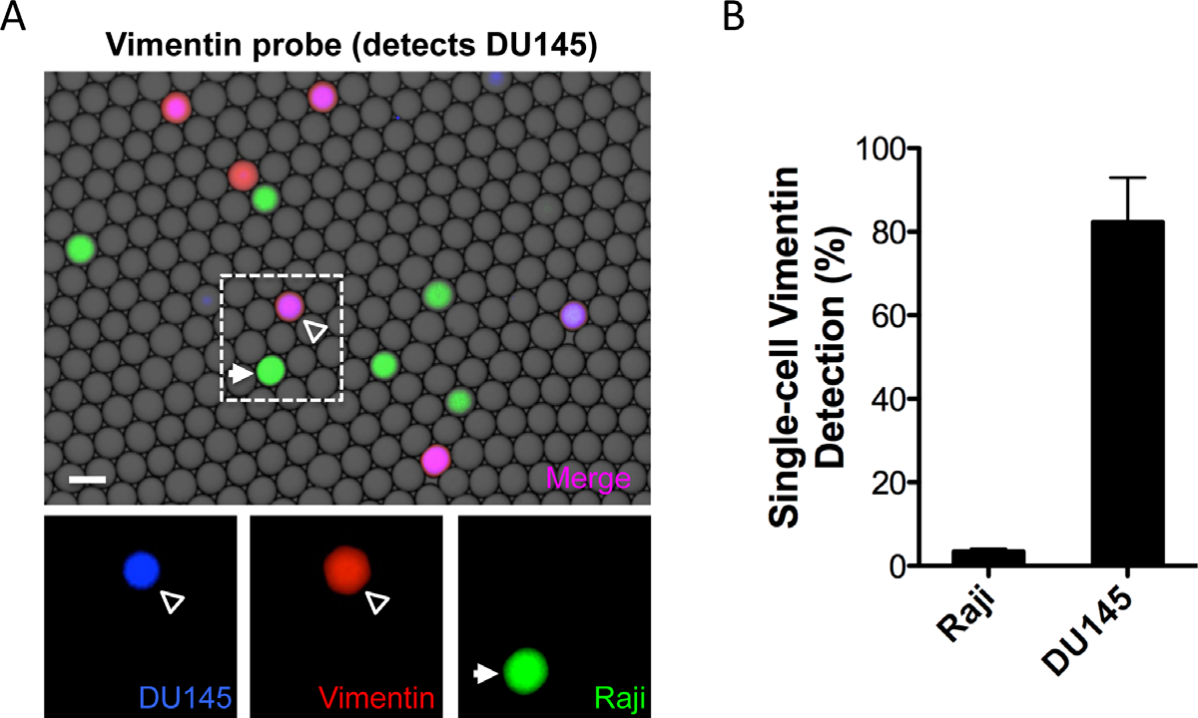
Single-cell vimentin TaqMan assays are specific for DU145 cancer cells. (**A**) Merged brightfield and fluorescence images showing amplified vimentin TaqMan probe (red), calcein violet DU145 (blue) and calcein green Raji (green) lysate in droplets. The presence of a purple-violet color indicates droplets where both DU145 calcein violet stain and HEX from the vimentin TaqMan probe were detected. Individual fluorescence channels from the dashed region are shown below. Scale bar is 100 μm. (**B**) Vimentin TaqMan detection rates of individual DU145 and Raji cells processed with the single-cell RT-PCR microfluidic workflow. Data was compiled from replicate experiments analyzed with a MATLAB script.

Calcein-labeled DU145 and Raji cells were mixed in roughly equal ratios and encapsulated in droplets for lysis. The droplets were then processed on our microfluidic system to prepare them for RT-PCR and add TaqMan reagents (8). Following droplet collection and thermocycling, the droplets were imaged on a fluorescence microscope to measure the intensities of the channels corresponding to the calcein dyes and HEX TaqMan probe (Figure 2A). The images were subsequently analyzed using a custom MATLAB script to measure the correlation between the two calcein dyes and the TaqMan probe signal (Supplementary Figure S2). This enabled us to determine the percentage of Raji and DU145 cells detected with the vimentin TaqMan reaction (Figure 2B). The detection rate for DU145 cells was 82.3% (+/− 15.1) and for Raji cells 3.4% (+/− 1.0). Although a low percentage of Raji cells appear to be vimentin-positive, correlation analysis between calcein violet and green cell stains indicates that the majority of these events occur from both Raji and DU145 cells being in the same droplet during cell encapsulation, a result of random Poisson loading (Supplementary Figure S2). We previously reported the ability to detect multiple transcript types in Raji cells with high efficiency, indicating that the extremely low number of Raji cells determined to express vimentin is not an artifact of reduced RT-PCR efficiency in the presence of Raji cell lysate (8). Together, these results demonstrate that vimentin mRNA is a specific biomarker for DU145 cells compared to Raji cells, and that by interrogating for vimentin we will be able to identify and recover these cells out of a heterogeneous population.

### Enrichment of DU145 cells out of a mixed population with PCR-activated cell sorting

**Figure 3.**
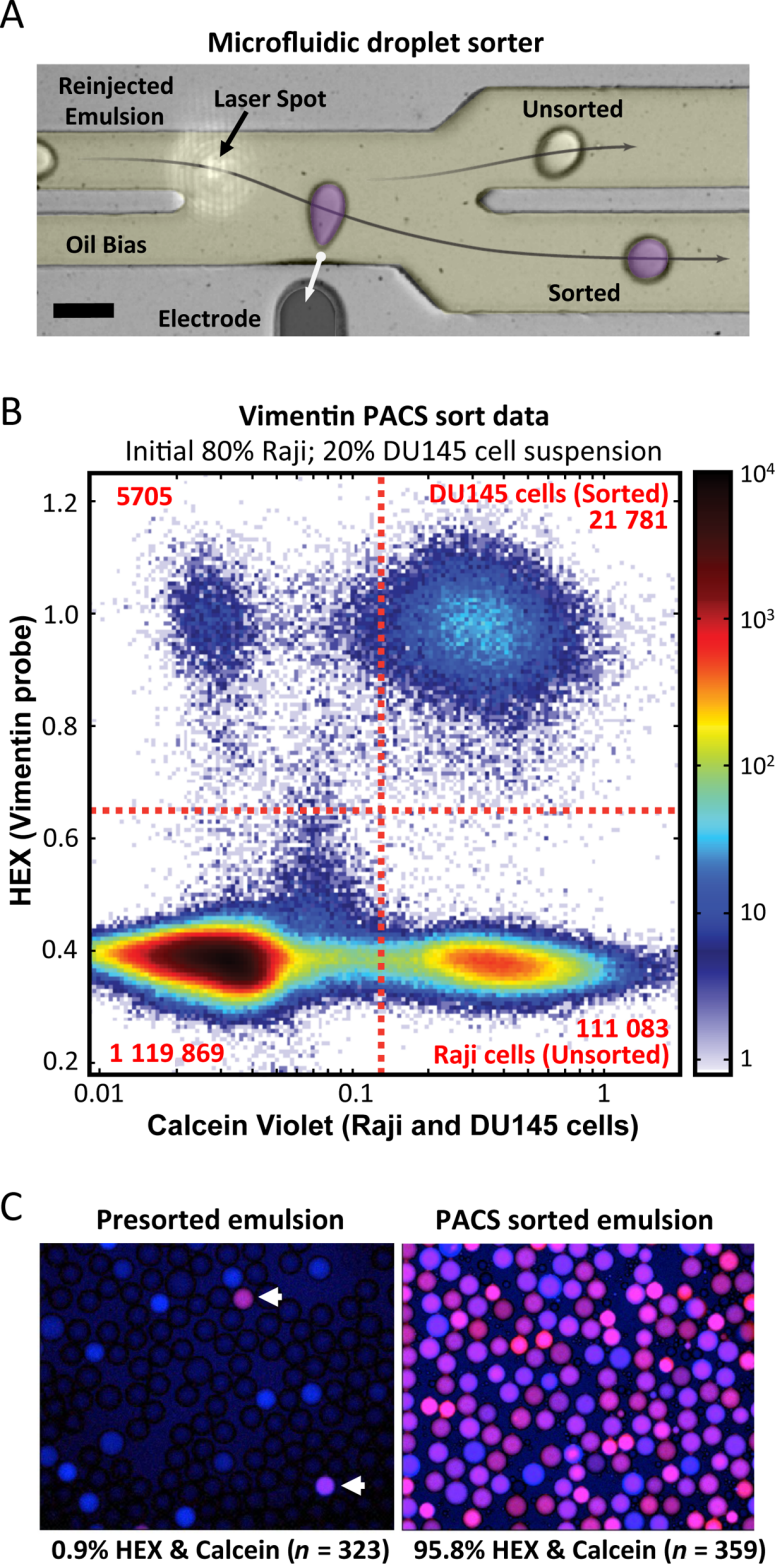
Ultrahigh-throughput detection and sorting with single-cell vimentin RT-PCR reactions. (**A**) Photograph of the dielectrophoretic microfluidic sorter. Reinjected emulsion entered the device from the left and was interrogated for fluorescence at the laser spot. A voltage was applied to the sorting electrode when a droplet was positive for both calcein and HEX above the specified thresholds. This pulled the specified droplets into the lower channel for collection. Scale bar is 100 μm. (**B**) Scatterplot diagram of single-cell RT-PCR sorted droplets showing the calcein violet cell stain fluorescence used to mark Raji and DU145 cells on the x axis and HEX fluorescence from the TaqMan positive reactions on the y axis. Dashed red lines indicate where the sorting thresholds were applied. Only droplets in the upper right quadrant were selected for sorting. This PACS data was generated from an initial 80% Raji and 20% DU145 heterogeneous cell suspension. (**C**) Presorted and sorted droplets were imaged to evaluate sorting efficiency. Arrowheads in the presorted emulsion image point to two droplets positive for both calcein and HEX. The rest of the droplets are either empty or only calcein positive. Nearly all of the droplets following sorting are positive for both calcein and HEX.

In addition to detecting cells based on nucleic acid analysis, the goal of PACS is also to recover the lysates of the positive cells. To demonstrate this capability, we prepared another sample in which DU145 cells were spiked into Raji cells at 20 and 80%, respectively. The cells were then labeled with calcein violet, which acted as a fluorescent indicator for droplets that originally contained a live cell; this is a critical internal control that allows us to identify false positive droplets undergoing amplification due to the presence of vimentin transcripts but that did not contain a single cell (‘digital background’). Following staining, cells were encapsulated, lysed and run through the RT-PCR preparation device, taking ∼4 h. The droplets were collected, thermocycled, stored overnight at 4°C, and sorted the following day. During sorting, we gated to recover droplets containing cell lysates positive for vimentin expression. This was accomplished by discarding all droplets which were below the gating thresholds for either the HEX or calcein fluorescent signals (uncolored, Figure 3A) and recovering all droplets above the thresholds for *both* signals (pseudo-colored purple, Figure 3A).

During sorting, we also collected statistics on the droplet fluorescence. A scatter plot of HEX versus calcein fluorescence reveals that we interrogated over 132 000 single cells and performed over 1.2 million droplet RT-PCRs, Figure 3B. The dashed red lines demarcate the sorting thresholds used to recover positive droplets. Of droplets containing cell lysate, 16.4% were also positive for TaqMan fluorescence (upper-right quadrant, Figure 3B). This measured value is in good agreement with the number of DU145 cells expected (16.5%) based on the controlled spike-in value (20%) and the detection rate independently measured in the previous experiment (82.3%, Figure 2B). A small fraction of droplets were devoid of calcein stain but nevertheless exhibited TaqMan signal. We have observed and reported this previously and attribute it to free vimentin transcripts released into suspension during cell encapsulation, which is likely due to inevitable cell death during this step (8). These ‘digital background’ droplets are discarded by the sorter, since they fall below the cell stain threshold (vertical dashed line, Figure 3B).

To confirm the function of the sorter and appropriate selection of sorting gates, we examined the sorted droplets and a small portion of the original presorted emulsion (Figure 3C). A scatterplot of the HEX and calcein fluorescence values revealed that 95.8% of positively-sorted droplets had significant calcein and HEX fluorescence (Supplementary Figure S3). Conversely, only 0.9% of presorted droplets were positive for both signals. This constitutes a more than 100-fold increase in the double-positive droplet ratio following sorting, and confirms the ability of PACS to enrich specific cells from a heterogeneous population.

### Genetic analysis of PACS-sorted cancer cells

**Figure 4.**
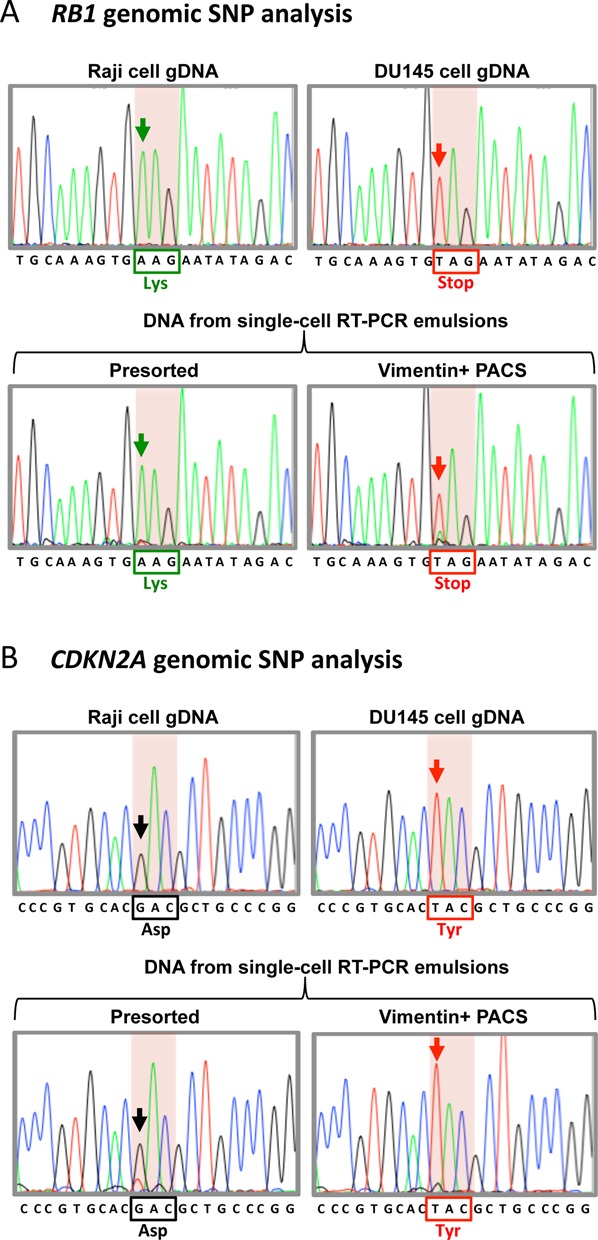
Sanger sequencing of DU145 enriched genomic DNA following sorting based on vimentin mRNA detection. (**A**) A portion of the *RB1* locus was amplified from genomic DNA isolated from individual cell lines and Sanger sequenced as a control (top two sequences). Raji cell *RB1* encodes for a lysine at amino acid position 715 (black box). DU145 genomic DNA has a nonsense mutation at this position (red box). Sequencing of genomic DNA amplified from droplets prior to vimentin-positive PACS sorting (Presorted) produces a Raji cell sequence with lysine at position 715. This is expected based on the initial encapsulation of a 90% Raji cell and 10% DU145 cell suspension. Following vimentin-positive droplet sorting (Vimentin+ PACS), sequencing shows that the genomic DNA is dramatically enriched for the DU145-specifc stop codon. (**B**) Sequencing of *CDKN2A* amplicons from control cell genomic DNA (top two sequences). Amino acid 84 is mutated from an aspartic acid to a tyrosine in DU145 cells (red box). Sequencing of the presorted single-cell RT-PCR emulsion DNA yields a Raji-specific aspartic acid. Following vimentin-positive PACS sorting, the genomic DNA is enriched for tyrosine encoding sequences. Arrows indicate the position of the SNPs.

A major advantage of PACS over FISH-FC is that it does not require chemical fixation, enabling facile analysis of nucleic acids recovered with sorting. To more thoroughly characterize the ability of PACS to specifically sort target cells out of a heterogeneous population and sequence the recovered material, we sorted a suspension containing 10% DU145 and 90% Raji cells. DU145 cells have genetic mutations in two commonly mutated tumor suppressor genes, *RB1* and *CDKN2A*, which likely contribute to the transformation of this prostate cancer cell line (25,26). These two mutations are homozygous SNPs residing at genetically unlinked genomic loci and are not found in Raji cells (upper panels, Figure 4A,B); consequently, they represent clear genetic biomarkers with which to estimate the fraction of DU145 and Raji DNA in the recovered material.

In this experiment, 92 996 individual cells were analyzed, of which 10.8% (10 099) were positive for vimentin RT-PCR and cell viability stain. To perform the genomic analysis, we isolated DNA from the pre- and post-sorted emulsions. Purified genomic DNA from a total of 1326 (∼13% of total droplets positively sorted) was used to amplify *RB1* and *CDKN2A*, and the SNP regions for both genes were analyzed by Sanger sequencing (Figure 4). In the presorted emulsion, both *RB1* and *CDKN2A* contain Raji SNP sequences and only a weak DU145-specific nucleotide peak, reflecting the relatively minor (10%) contribution of DU145 DNA in this emulsion. By contrast, after sorting, sequences associated with DU145 cells dominate, with only trace Raji sequences still present, as illustrated in Figure 4A,B, lower chromatograms. This shows that PACS can enrich the genotype of an initially undetectable cancer cell population by sorting the heterogeneous cells based on expression of a cancer-associated gene.

**Figure 5.**
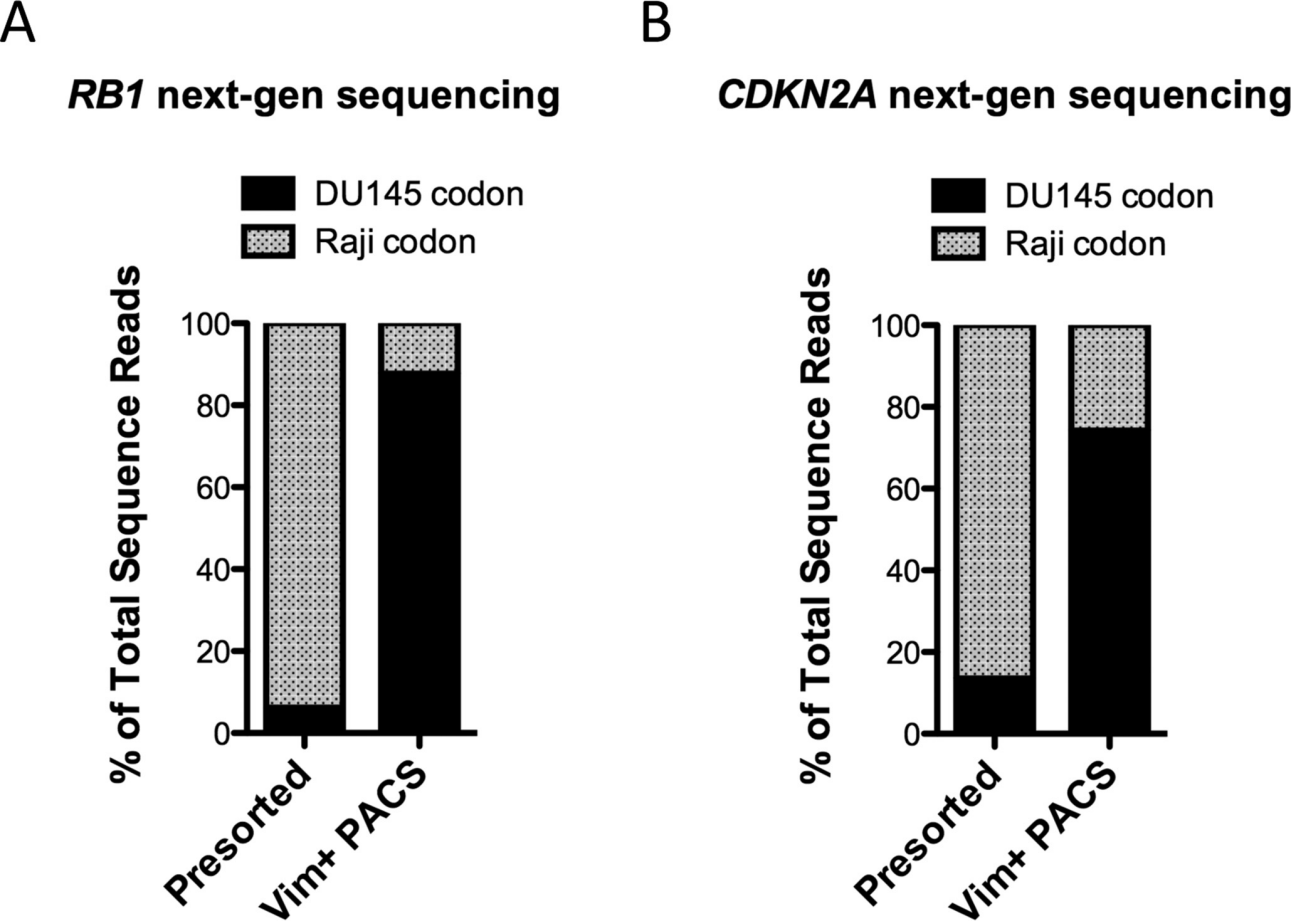
Quantitative analysis of PACS genome enrichment with next-generation sequencing. Analysis of *RB1* and *CDKN2A* genomic loci for the presence of DU145-specific SNPs. Sequencing libraries from *RB1* and *CDKN2A* amplicons were generated using Nextera XT reagents. (**A**) Quantitative analysis of *RB1* sequence reads demonstrated that the DU145-specific nonsense mutation, AAG to TAG (see Figure 4), was found in 6.2% of the sequence reads generated from presorted cell lysate. Following PACS sorting (Vim+ PACS) the presence of this mutation relative to the Raji-specific codon was enriched to 87.7%. (**B**) Similar data was obtained upon sequence analysis of *CDKN2A* amplicons generated from presorted and Vim+ PACS sorted cell lysate. The DU145-specific missense mutation, GAC to TAC (see Figure 4), went from comprising 13.5% of the sequence reads to 74.2% of the sequence reads upon PACS enrichment. More than 15 000 sequence reads were analyzed for each of the four samples.

The Sanger sequencing chromatograms provide a semi-quantitative measurement of DU145 enrichment, but to obtain a more accurate measurement, we turned to next-generation sequencing, which allowed us to measure the exact proportion of SNPs associated with the two different cell types in the sorted pool. We generated Nextera libraries from the *RB1* and *CDKN2A* amplicons obtained in the previous experiment. Following next-generation sequencing, we measured the percentage of reads containing SNPs from the two different cell types (Figure 5). In the presorted emulsion, for *RB1*, DU145-specific codons comprised 6.2% of the total reads. Following vimentin-positive PACS, this codon variant was 87.7% of all reads. Similar results were obtained for the *CDKN2A* locus, with DU145 codons accounting for 13.5% of total reads pre-sorting and 74.2% post-sorting. The small differences between the *RB1* and *CDKN2A* enrichment values may be due to bias introduced during sequencing library preparation with Nextera and PCR. These results are consistent with the expected SNP percentages in the original 10% DU145 and 90% Raji cell suspension, and provide a more quantitative validation of successful enrichment of DU145 cells with PACS.

### Gene expression analysis of PACS-sorted cells

**Figure 6.**
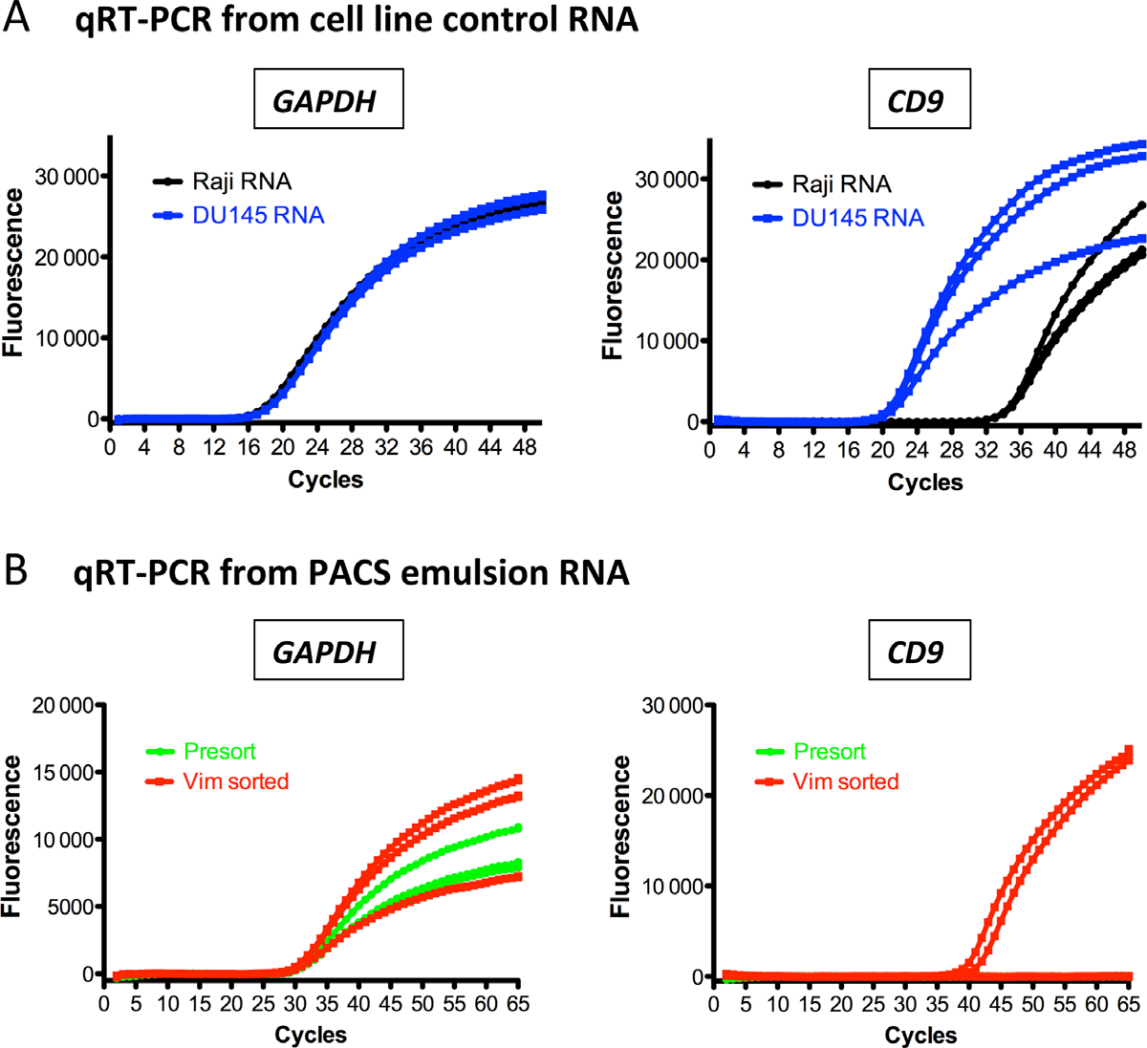
mRNA expression analysis following PACS enrichment. (**A**) qRT-PCR amplification curves from GAPDH or CD9 performed on cell line isolated total RNA control samples. CD9 was expressed significantly higher in DU145 cells than in Raji cells. (**B**) RNA from presorted and vimentin-positive PACS (Vim sorted) droplets was analyzed for CD9 expression following normalization of input levels with GAPDH. CD9 was only detected in the vimentin-positive sorted droplets indicating that PACS enriched for DU145 expressed transcripts. Three replicate amplification curves are shown for each qRT-PCR experiment.

In many applications of cell sorting, the goal is to recover a specific subset of cells and subject that subset to detailed molecular analysis. As we have shown, PACS enables sequencing of the genomes of sorted cells, but in many instances it would also be valuable to analyze the sorted cell transcriptomes. To investigate the ability of PACS to enable downstream gene expression analysis of sorted cells, we examined RNA recovered from vimentin-positive droplets for the enrichment of transcripts differentially expressed in DU145 cells compared to Raji cells. Analysis of control RNA isolated from individual Raji and DU145 cells demonstrated that CD9 expression is more than 16 000× higher in DU145 cells than in Raji cells when normalized to GAPDH expression (Figure 6A). Following vimentin-positive PACS on the DU145 and Raji cell suspension, RNA recovered from 4659 TaqMan sorted droplets was divided and used for GAPDH and CD9 qRT-PCR replicates (Figure 6B). RNA input for presorted and PACS sorted samples were normalized with GAPDH controls. CD9 was clearly detected in PACS sorted droplets and absent from the presorted emulsion. This indicates that downstream gene expression analysis on lysates recovered with PACS is feasible. An additional benefit of PACS is that it uses RT-PCR to detect the cell types of interest; implementation of transcriptome-wide first strand cDNA synthesis in this step should facilitate transcriptome analysis, including with RNA-Seq and microarrays.

### PACS can be used to detect and sort cells with genome targeted TaqMan assays

Detecting a single copy of a DNA sequence in a cell's genome requires an assay capable of single molecule sensitivity. To investigate the feasibility of identifying cells based on sequences present in their genome, and to test the sensitivity of TaqMan reactions performed with PACS, we targeted the *SRY* gene found on the human Y chromosome (14). DU145 prostate cancer cells are male in sex and therefore harbor a single copy of the *SRY* gene in their genome. Although the *SRY* probe is targeted to an exon, we omitted reverse transcriptase from the reaction and included RNase A to ensure genomic *SRY* DNA specificity. To further control for specificity of the TaqMan reaction we included female HEK293 cells, lacking a Y chromosome and the *SRY* gene, as a negative control cell population in the single-cell PCR workflow (27).

**Figure 7.**
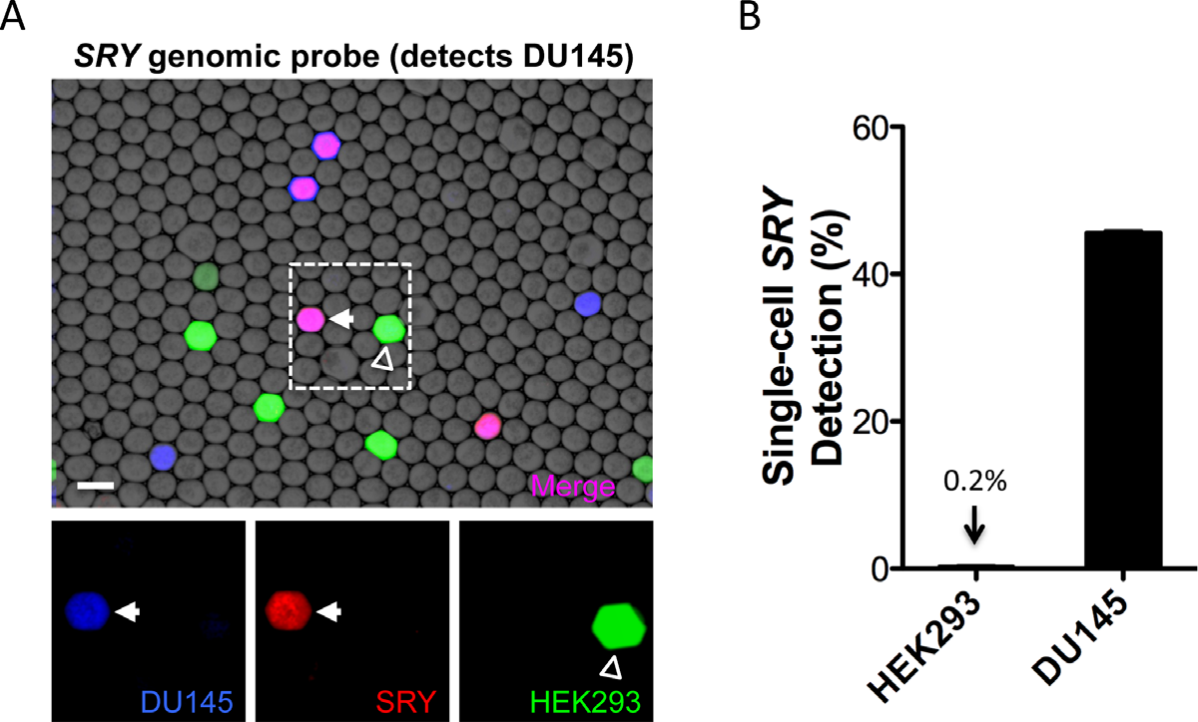
Single-cell genomic SRY TaqMan assays are specific for male DU145 cells. (**A**) Merged brightfield and fluorescence images showing amplified *SRY* TaqMan probe (red), calcein violet DU145 (blue) lysate and calcein green HEK293 (green) lysate in droplets. The presence of a purple-violet color indicates droplets where both DU145 calcein violet stain and HEX from the *SRY* TaqMan probe were detected. Individual fluorescence channels from the dashed region are shown below. Scale bar is 100 μm. (**B**) *SRY* TaqMan detection rates of individual DU145 and HEK293 cells processed with the single-cell PCR microfluidic workflow. The *SRY* reaction detection rate for DU145 cells was 45.6% (+/− 0.4%) and the detection rate for HEK293 cells was 0.2% (+/− 0.3%). Data was compiled from replicate experiments analyzed with a MATLAB script.

DU145 cells were labeled with calcein violet and HEK293 cells were labeled with calcein green to enable us to correlate *SRY* amplification with cell type. Mixed DU145 and HEK293 cell suspensions were encapsulated in droplets and processed with the microfluidic workflow to prepare the single-cell *SRY* TaqMan reactions for thermocycling. Following thermocycling fluorescence microscopy was used to examine the droplets for cell stains and *SRY* probe detection (Figure 7A). Analysis of the fluorescent droplet data showed that *SRY* amplification was highly specific for DU145 cells (Figure 7B and Supplementary Figure S4). These results demonstrate that PACS provides the sensitivity to identify cells based on sequences in their genome.

**Figure 8.**
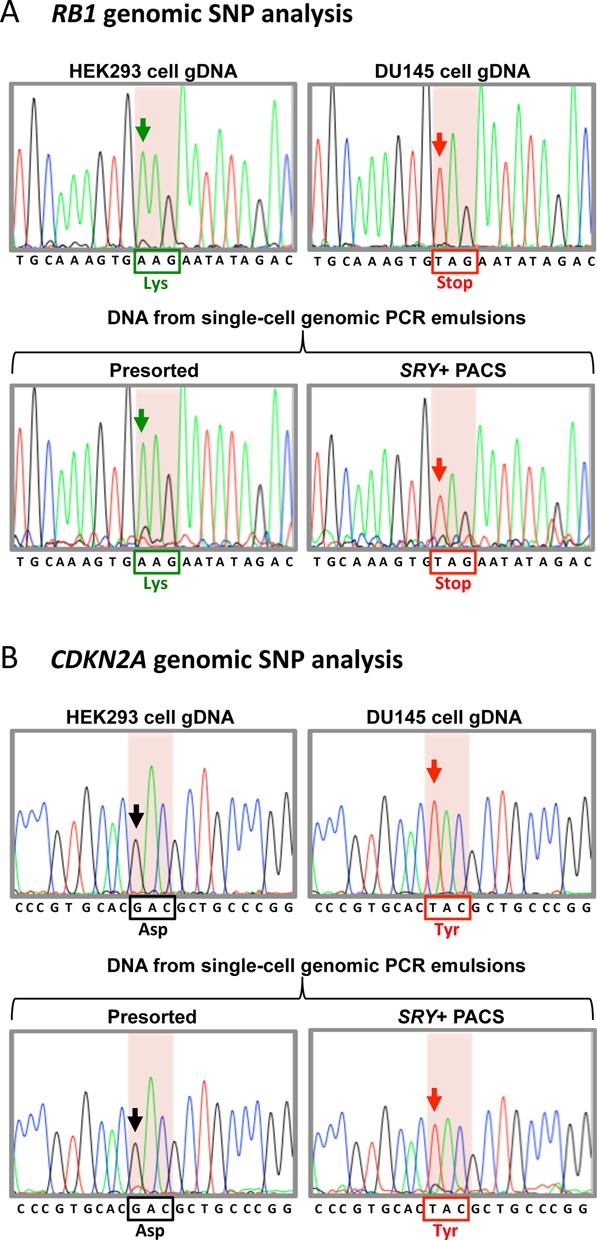
Enrichment of DU145-specific genomic SNPs following sorting based on Y chromosome DNA detection. (**A**) A portion of the *RB1* locus was amplified from genomic DNA isolated from individual female HEK293 and male DU145 cell lines and Sanger sequenced as a control (top two sequences). Similar to the results shown in Figure 4, DU145 genomic DNA contains a nonsense mutation at amino acid position 715 (red box). Sequencing of genomic DNA amplified from droplets prior to *SRY*-positive PACS sorting (Presorted) produces a HEK293 cell sequence with a lysine at position 715. This lysine codon predominates due to the fact that the initial cell suspension contained 90% HEK293 cells. Following *SRY*-positive droplet sorting (*SRY*+ PACS), sequencing shows that the genomic DNA is enriched for the DU145-specifc stop codon. (**B**) Sequencing of *CDKN2A* amplicons from control cell genomic DNA (top two sequences). Amino acid 84 is mutated from an aspartic acid to a tyrosine in DU145 cells (red box). Sequencing of the presorted single-cell RT-PCR emulsion DNA yields a HEK293-specific aspartic acid. Following *SRY*-positive PACS sorting, the genomic DNA is enriched for tyrosine encoding sequences. Arrows indicate the position of the SNPs.

We next investigated the ability to perform the complete PACS workflow using the genome targeted *SRY* TaqMan assay. A mixed cell suspension containing 90% female HEK293 cells and 10% male DU145 cells was calcein violet stained, processed with the single-cell PCR microfluidic workflow and thermocycled. The thermocycled droplets were then reinjected into the detection and sorting microfluidic device to sort based on the dual presence of calcein and *SRY* TaqMan assay fluorescence. A scatterplot of the *SRY* PACS sort data is shown in Supplementary Figure S5. 1224 calcein and *SRY* dual-positive droplets were sorted and collected for downstream enrichment analysis. To examine whether our DU145 enrichment strategy was successful, we employed the *RB1* and *CDKN2A* DU145-specific SNP assays, discussed earlier, on genomic DNA from presorted and *SRY*-positive sorted droplets. Twenty five percent of the genomic DNA isolated from the 1224 PACS-sorted droplets was used in each *RB1* and *CDKN2A* amplification reaction prior to Sanger sequencing. The results from this analysis clearly demonstrate the ability to enrich for DU145-specific genomic DNA SNPs at both the *RB1* and *CDKN2A* loci following PACS targeting *SRY* (Figure 8). These data demonstrate that the PACS workflow can be used to enrich cell lysate based not only on the presence of mRNA transcripts, but also unique sequences present in single-cell genomic DNA.

## DISCUSSION

PACS represents a completely novel approach to cell sorting and is valuable for addressing a variety of questions in basic biology and human disease. Until now, the principal challenge to implementing PACS has been the potent inhibitory effect of mammalian cell lysate on PCR in picoliter volumes and the difficulty of overcoming inhibition in a manner that is scalable to the analysis of large numbers of cells. Our novel microfluidic workflow prepares single-cell lysates for PCR in microfluidic droplets and enables the use of TaqMan reactions upon which to trigger cell sorting. The use of single-cell TaqMan reactions not only provides extremely specific detection of cells, but should also be compatible with analysis of non-coding RNAs, transcript splice variants, genetic mutations and other nucleic acid biomarkers undetectable with antibodies.

Droplet-based digital PCR reactions are extremely sensitive, enabling the detection of single transcripts or copies of DNA (21,28). We show that PACS provides a similar level of sensitivity by demonstrating the detection of single-copy Y chromosome DNA from individual cells. This capability should enable sorting of cells by detecting rare nucleic acids within cells such as genomic mutations or low copy number transcripts. Although it is challenging to fully explore the limits of mRNA detection and differential resolution with PACS, this capability is likely to be robust as well. In support of this, we have performed multiplexed TaqMan reactions and obtained high efficiency detection of multiple transcript types in single-cells with PACS (Supplementary Figure S6). We have also previously published the ability to use TaqMan probes targeting transcripts other than vimentin to perform high specificity differential detection of mixed cell populations processed with our microfluidic single-cell RT-PCR workflow (8). Despite the robustness of the PACS single-cell reactions, the detection of cells is based on an endpoint TaqMan fluorescent signal and is thus only semi-quantitative in nature. Consequently, PACS is currently best suited for identifying cells with unique nucleic acid sequences or having substantial differential transcript expression. The ability to differentially resolve two populations of cells will also be influenced by the exact nature of the heterogeneous distribution of the PACS-targeted nucleic acid biomarker within single cells.

PACS is capable of analyzing single cells with RT-PCR at unprecedented throughput. In this study, we report the RT-PCR analysis and sorting of 132 000 cells in a single experiment. Although this level of throughput does not yet approach the sorting rate for state-of-the-art FACS instruments, it represents a more than 500-fold improvement over other methods for implementing mammalian single-cell RT-PCR (7,29–31). Furthermore, our method is scalable and holds the potential for at least a 10-fold increase in single-cell throughput. This increase in throughput can be accomplished by improving the cell encapsulation efficiency to load more of the droplets with single cells and improvements to the microfluidic devices enabling higher droplet velocities (32). Even at the current throughput of >100 000 cells per experiment, PACS is uniquely suited for analyzing heterogeneous cell populations, including immune cells, large tumors and even circulating tumor and fetal cells, especially when combined with cell pre-enrichment or depletion strategies. Such strategies can even be integrated directly into the PACS workflow by sorting cells based on antibody fluorescence at the time of encapsulation.

A critical advantage of PACS is its ability to recover nucleic acids for downstream analysis; this will make it valuable for addressing numerous research questions. As we have shown, the genomes of PACS-sorted cells can be sequenced in a targeted fashion and, with the implementation of appropriate library preparation steps downstream, whole genome sequencing should be possible too. In this way, PACS can be used to not only identify and enumerate cells based on virtually any nucleic acid biomarker for a given behavior or cell state, but can subsequently be used to study the genetic drivers behind that behavior. In addition to genomic analysis, we have also shown gene expression analysis of PACS-recovered cells, which will aid in the investigation of the regulatory networks and effector proteins behind cell states and behaviors. In future work, we will investigate performing transcriptome-wide reverse transcription during the TaqMan RT-PCR step to prepare cDNA libraries from single cells in preparation for downstream sequencing. This strategy should simplify single cell sequencing and can be combined with in-droplet barcoding to enable the parallel sequencing of thousands of single cells (33).

The ability to sort cells without antibodies makes sorting easier in many instances and enables sorting in instances not presently possible, because the identifying biomarker is a nucleic acid and undetectable with current methods. For example, PACS should allow the detection and isolation of human cells infected with a latent virus or the sorting of tumor cells based on expression of microRNAs that correlate with metastasis or contribute to the pathology of the disease. Moreover, PACS is, in its most general form, a sorter of nucleic acids, whether those nucleic acids exist behind a cell membrane, viral capsid or are free to diffuse in solution. We therefore anticipate that in addition to sorting microbes and viruses, PACS will also be used to sort long genomic fragments, or even whole chromosomes.

## SUPPLEMENTARY DATA

Supplementary Data are available at NAR Online.

SUPPLEMENTARY DATA
