# Supplementary material for: Identification and genetic analysis of cancer cells with PCR-activated cell sorting
Source: Nucleic Acids Res. 2014 Jul 16;42(16):e128. doi: 10.1093/nar/gku606 (PMC4176366; doi:10.1093/nar/gku606)
Supplement: SUPPLEMENTARY DATA [file supp_42_16_e128__index.html]

Identification and genetic analysis of cancer cells with PCR-activated cell sorting — Identification and genetic analysis of cancer cells with PCR-activated cell sorting — SUPPLEMENTARY DATA 

# Identification and genetic analysis of cancer cells with PCR-activated cell sorting

## SUPPLEMENTARY DATA

**Files in this Data Supplement:**

- SUPPLEMENTARY DATA
