# Supplementary material for: Identification and genetic analysis of cancer cells with PCR-activated cell sorting
Source: Nucleic Acids Res. 2014 Jul 16;42(16):e128. doi: 10.1093/nar/gku606 (PMC4176366; doi:10.1093/nar/gku606)
Supplement: SUPPLEMENTARY DATA [file supp_gku606_nar-00979-met-k-2014-File010.pdf]

SUPPLEMENTARY DATA

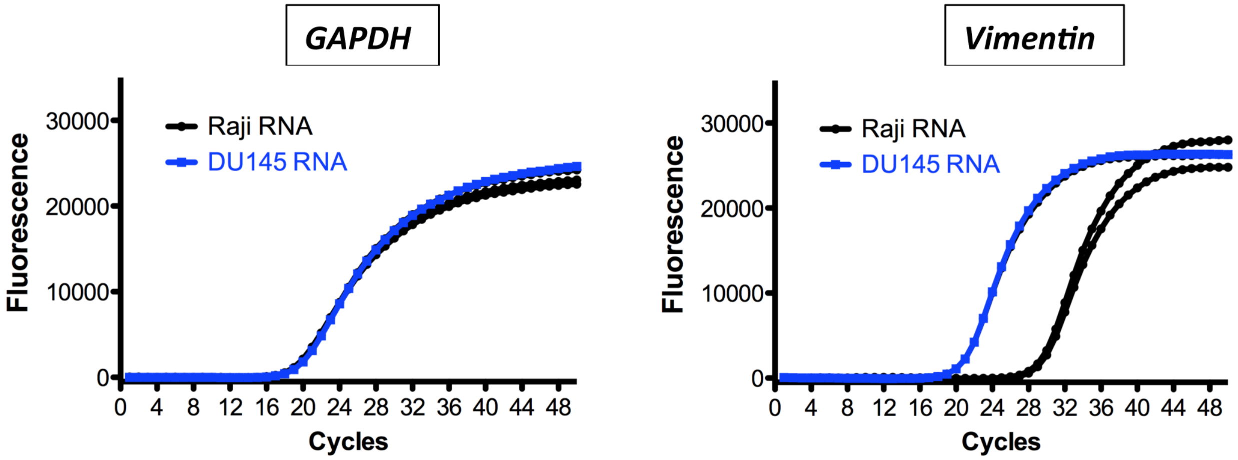

Supplementary Figure S1. *Analysis of relative vimentin mRNA expression levels between DU145 and Raji cells.* qRT-PCR amplification curves from GAPDH or vimentin performed on 50 ng/reaction of cell line isolated total RNA. GAPDH was used to control for input total RNA amount and normalize vimentin expression levels. Vimentin was expressed significantly higher in DU145 cells (average Ct = 21.36) than in Raji cells (average Ct = 29.93). Two replicate amplification curves are shown for each qRT-PCR experiment.

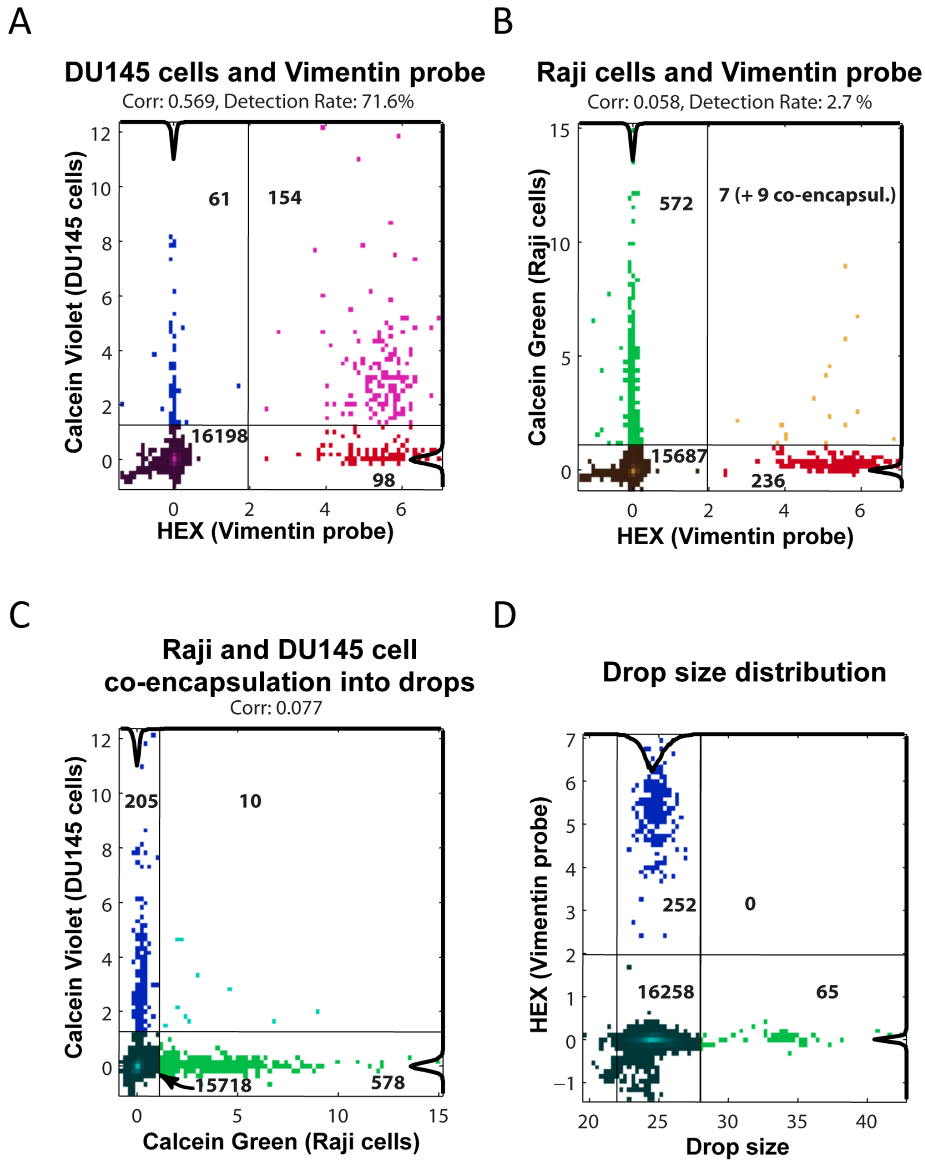

Supplementary Figure S2. *MATLAB generated scatterplots of drop fluorescence obtained from microscope images demonstrate vimentin probe specificity for DU145 cell lysate.* (A) Vimentin fluorescence correlates well with DU145 cell lysate marked by calcein violet stain. (B) Correlation between vimentin RT-PCR and Raji cell lysate marked with calcein green stain is weak and the majority of events detected were due to co-encapsulation of Raji and DU145 cells. In 9 of 16 droplets where vimentin was detected with Raji cell lysate, DU145 lysate was also detected. (C) Raji and DU145 co-encapsulation is driven by Poisson statistics and is quite rare (1.3% of cell containing droplets). (D) Analysis of droplet size distribution indicates a high degree of emulsion stability.

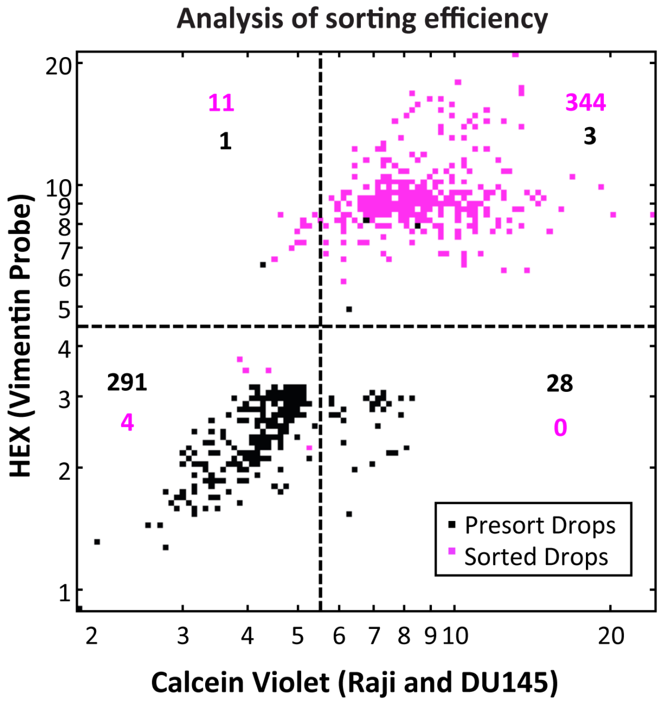

Supplementary Figure S3. *PACS droplet sorting efficiency is robust.* Scatterplot analysis of fluorescent images taken of presorted (black plots) and PACS sorted droplets (violet plots). As expected, the presorted droplets are mostly empty and lack either calcein or vimentin-positive TaqMan HEX signal. Only 3 of these droplets were positive for both signals (upper right quadrant). In contrast, almost all (95.8% n=359) of the droplets that were PACS workflow sorted based on the presence of both cell lysate (calcein) and TaqMan signal (HEX) are indeed positive. This analysis demonstrates the high fidelity nature of the microfluidic sorting device employed in the PACS workflow.

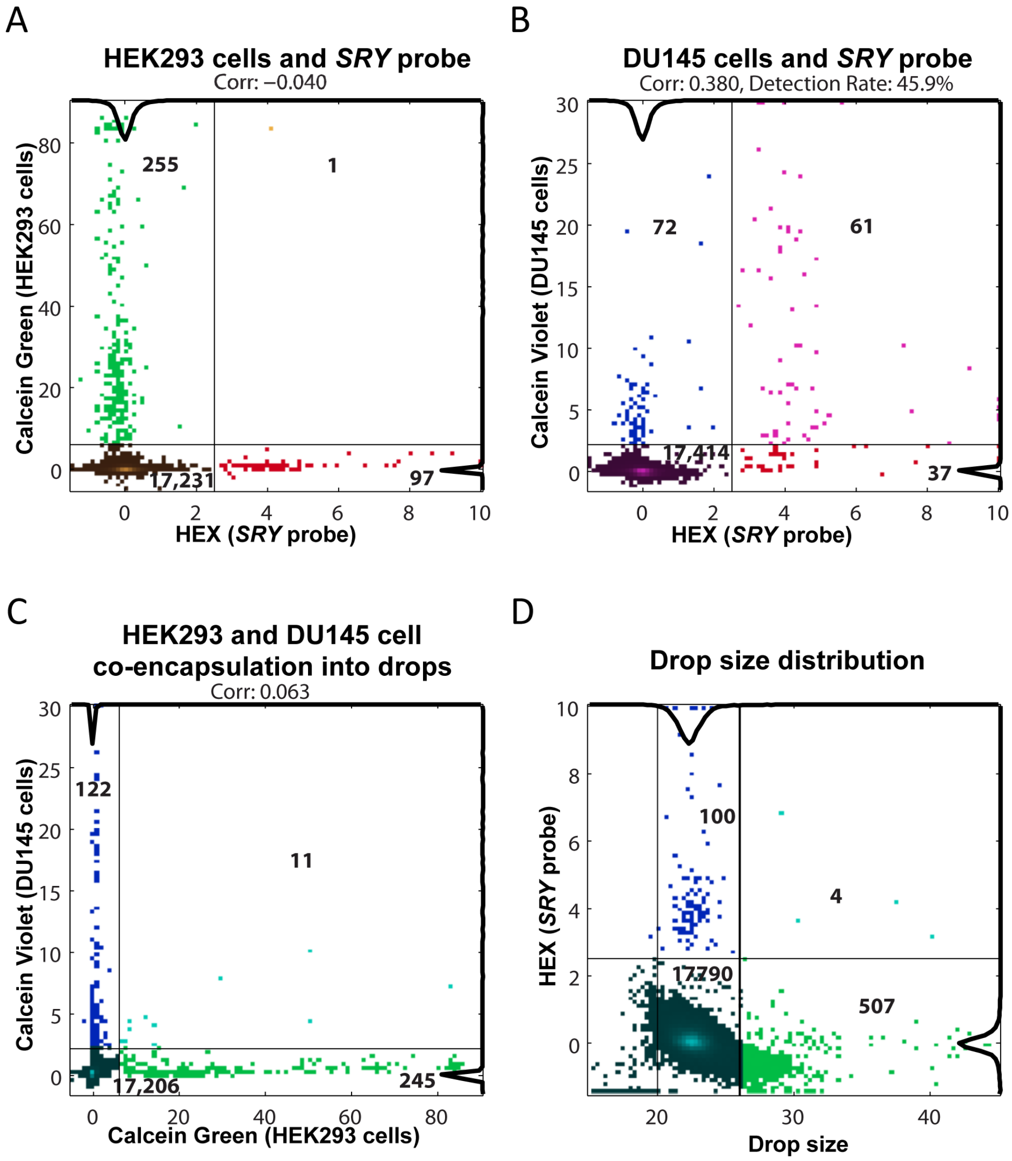

Supplementary Figure S4. *MATLAB generated scatterplots of drop fluorescence obtained from microscope images demonstrate SRY probe specificity for DU145 cell lysate.* (A) Correlation between *SRY* PCR and HEK293 cell lysate, marked with calcein green stain, was extremely rare. Only one double positive was detected from two replicate experiments and this was likely due to co-encapsulation with a DU145 cell or a cell-free chromosomal fragment during drop making (B) *SRY* fluorescence correlates well with DU145 cell lysate, marked by calcein violet stain. (C) HEK293 and DU145 co-encapsulation events. (D) Analysis of droplet size distribution indicates a high degree of emulsion stability.

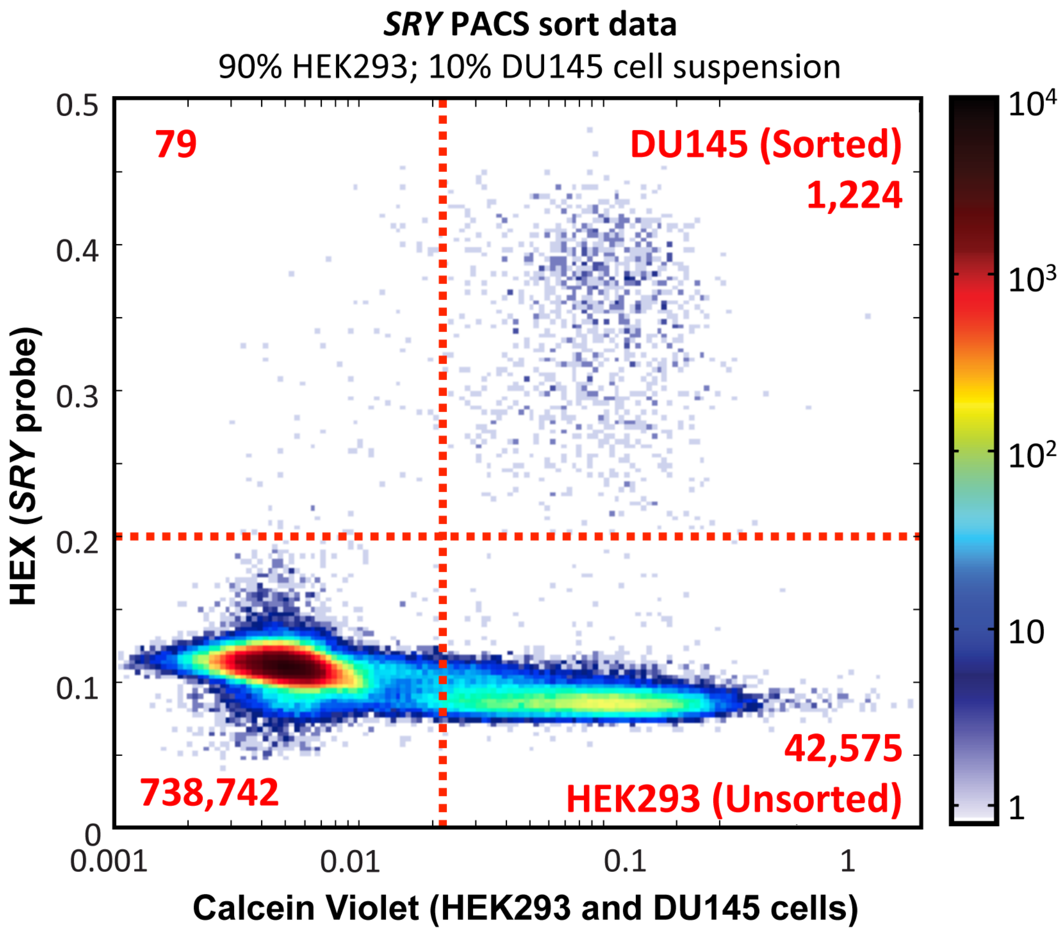

Supplementary Figure S5. *Detection and sorting of DU145 cancer cells using a genomic SRY TaqMan assay.* Scatterplot diagram of single-cell RT-PCR sorted droplets showing the calcein violet cell stain fluorescence used to mark HEK293 and DU145 cells on the x axis and HEX (*SRY* probe) fluorescence from the TaqMan positive reactions on the y axis. Dashed red lines indicate where the sorting thresholds were applied. Only droplets in the upper right quadrant (DU145 cells) were selected for sorting. This PACS data was generated from an initial 90% HEK293 and 10% DU145 heterogeneous cell suspension. Sorted droplets shown in this scatterplot were used for subsequent downstream sequencing to demonstrate successful DU145 lysate enrichment following PACS (Fig. 8).

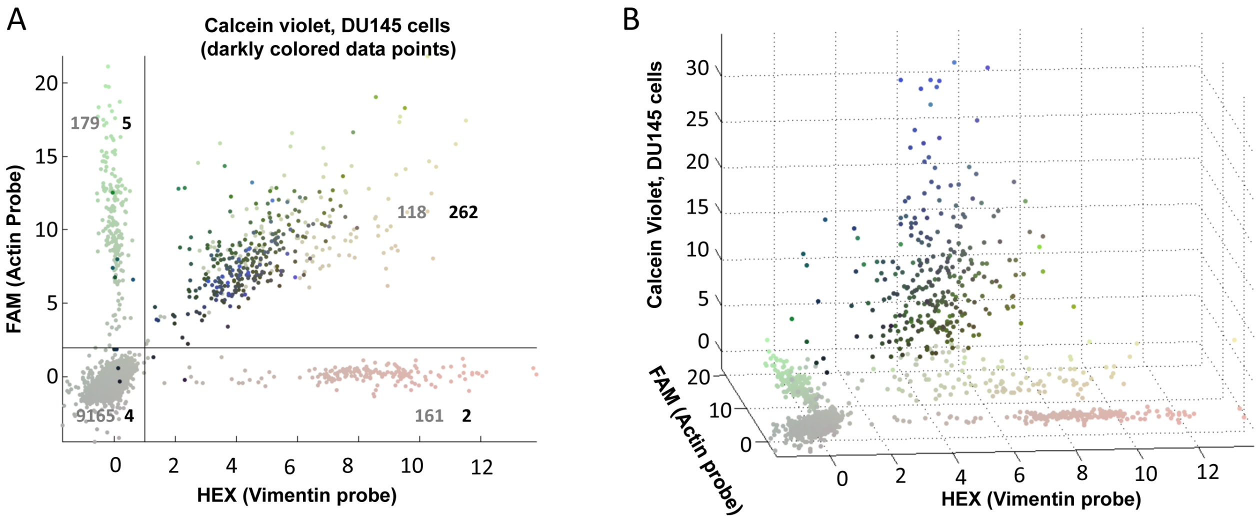

Supplementary Figure S6. *Efficient multiplexing with droplet-based single-cell RT-PCR TaqMan assays.* DU145 cells were examined for the simultaneous expression of actin and vimentin transcripts using a single-cell multiplexed TaqMan assay. Data from fluorescent images taken of the droplets following microfluidic single-cell RT-PCR preparation and thermocycling is shown in scatterplots. (A) Two-dimensional plot of actin and vimentin TaqMan probe fluorescence in drops. Drops that also contain DU145 cell lysate, determined by calcein violet staining DU145 cells, are plotted as the darker data points. Drops without calcein fluorescence appear faded. Actin and vimentin were simultaneously detected with the multiplexed reaction in 96.0% (n=273) of all drops containing DU145 lysate. The high detection rate with this multiplex reaction indicates that the reactions are efficient and robust. (B) The same data presented in (A) is shown on a three-dimensional plot with calcein violet fluorescence represented by the vertical axis. Multiplex reactions such as this can be extremely useful in identifying and isolating unique cell types based on the correlation of multiple biomarkers with PACS.
